# Supplementary figures and images for: Icariin Alleviates Nonalcoholic Fatty Liver Disease in Polycystic Ovary Syndrome by Improving Liver Fatty Acid Oxidation and Inhibiting Lipid Accumulation
Source: Molecules. 2023 Jan 5;28(2):517. doi: 10.3390/molecules28020517 (PMC9861792; doi:10.3390/molecules28020517)

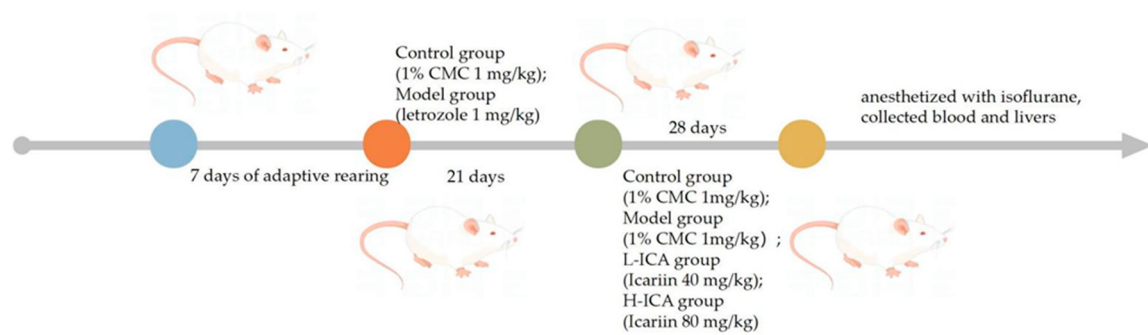

**Figure S1.** Flowchart describing the different groups.

Supplement: Supplementary file 1 [file molecules-28-00517-s001.zip › molecules-2096889-supplementary material.pdf]
